# Dissecting the Invasion-Associated Long Non-coding RNAs Using Single-Cell RNA-Seq Data of Glioblastoma

**DOI:** 10.3389/fgene.2020.633455

**Published:** 2021-01-11

**Authors:** Bo Pang, Fei Quan, Yanyan Ping, Jing Hu, Yujia Lan, Lin Pang

**Affiliations:** College of Bioinformatics Science and Technology, Harbin Medical University, Harbin, China

**Keywords:** single-cell RNA sequencing, glioblastoma, invasion, long non-coding RNA, survival

## Abstract

Glioblastoma (GBM) is characterized by rapid and lethal infiltration of brain tissue, which is the primary cause of treatment failure and deaths for GBM. Therefore, understanding the molecular mechanisms of tumor cell invasion is crucial for the treatment of GBM. In this study, we dissected the single-cell RNA-seq data of 3345 cells from four patients and identified dysregulated genes including long non-coding RNAs (lncRNAs), which were involved in the development and progression of GBM. Based on co-expression network analysis, we identified a module (M1) that significantly overlapped with the largest number of dysregulated genes and was confirmed to be associated with GBM invasion by integrating EMT signature, experiment-validated invasive marker and pseudotime trajectory analysis. Further, we denoted invasion-associated lncRNAs which showed significant correlations with M1 and revealed their gradually increased expression levels along the tumor cell invasion trajectory, such as VIM-AS1, WWTR1-AS1, and NEAT1. We also observed the contribution of higher expression of these lncRNAs to poorer survival of GBM patients. These results were mostly recaptured in another validation data of 7930 single cells from 28 GBM patients. Our findings identified lncRNAs that played critical roles in regulating or controlling cell invasion and migration of GBM and provided new insights into the molecular mechanisms underlying GBM invasion as well as potential targets for the treatment of GBM.

## Introduction

Glioblastoma (GBM) is the most common primary malignant brain tumor, comprising 16% of all primary brain and central nervous system neoplasms (Thakkar et al., 2014), with the average age-adjusted incidence rate of 3.2 per 100,000 population (Ostrom et al., 2015). Due to fast and invasive growth of the tumor, the current therapeutic option shows many limitations in its efficacy and almost all patients present the progression of the disease with a mean progression-free survival of 7–10 months (Stupp et al., 2005) and a 5-year survival rate of less than 10% (Yang et al., 2019). Though great endeavors have been performed in the past few decades, survival has not improved significantly (Wolf et al., 2019). Therefore, determining the factors which are associated with the invasion of glioblastoma is of great significance.

Apart from protein-coding genes (PCGs), long non-coding RNAs (lncRNAs), as one kind of important regulators in biological development and disease progression (Batista and Chang, 2013), were frequently reported to control the invasion and metastasis of diverse cancer types, including glioblastoma. For example, epigenetic silencing of LINC00632 could result in the CDR1as depletion, which promoted invasion *in vitro* and metastasis *in vivo* through a miR-7-independent, IGF2BP3-mediated mechanism in melanoma (Hanniford et al., 2020). The lncRNA-ATB was upregulated in hepatocellular carcinoma and further promoted the upregulation of ZEB1 and ZEB2 by competitively binding the miR-200 family, which finally induced epithelial-mesenchymal transition (EMT) and invasion (Yuan et al., 2014). The gain-of-function or loss-of-function experiments also validated the association of lncRNAs SChLAP1 and Zbtb7a with invasive prostate cancer (Prensner et al., 2013; Wang et al., 2013). Although these studies contributed to the understanding of tumor invasion, they mostly focused on few lncRNAs. Besides, utilizing traditional experiment techniques including bulk RNA sequencing also has limitations in revealing the molecular mechanisms underlying GBM invasion.

Instead, single-cell RNA sequencing (scRNA-seq) generates gene expression profiles at single-cell resolution (Tang et al., 2009), which has emerged as a powerful tool to comprehensively determine cellular states in healthy and diseased tissues (Hovestadt et al., 2019). It has been applied to subtly characterize the heterogeneity of diverse cancers and identify rare cell populations as well as key factors associated with tumorigenesis and progression (Chung et al., 2017; Li et al., 2017), which also provides an unprecedented chance to capture the important lncRNAs that participate in GBM invasion and precisely delineate their roles during GBM progression.

In the current study, we took advantage of scRNA-seq data to identify modules that showed significant overlap with differentially expressed genes (DEGs). We integrated multiple resources including EMT signatures, invasive markers and pseudotime analysis to determine the GBM invasion-associated lncRNAs and further validated our findings in an extra scRNA-seq data set. Finally, our results of the present study could provide new insights into pathological mechanism research and new therapeutic target of GBM invasion.

## Materials and Methods

### Quantification and Quality Control

The raw data for most of the analyses in this study were downloaded from the GEO database (GSE84465). This data was published by Darmanis et al. (2017) and included 3589 cells from four primary GBM patients (BT_S1, BT_S2, BT_S4, and BT_S6). The labels of malignant cells and normal cells were provided by the authors. Raw reads were mapped to the human genome (hg19) by Bowtie (version 1.1.1) (Langmead et al., 2009), and the gene expression levels were quantified as transcripts per million (TPM) using RSEM (version 1.2.28) (Li and Dewey, 2011) with the option estimate-rspd and default parameters. Log2 transformed TPM values with an offset of 1 were used to denote expression levels. We excluded low-quality cells with less than 100,000 aligned reads or with less than 2000 detected genes. We further discarded genes with the number of expressed cells less than 50. As a result, we retained 998 GBM cells and 2347 normal cells with 11520 PCGs and 1877 lncRNAs.

The processed data (GSE131928) for validation was downloaded from the GEO database, which contains 6863 GBM cells and 1067 normal cells from 28 patients. This data was published by Neftel et al. (2019). We excluded PCGs with less than 50 expressed cells or lncRNAs with less than 5 expressed cells. Finally, we retained 11441 PCGs and 585 lncRNAs.

### Differential Expression Analysis and Functional Annotation

We used the MAST software package (version 1.14.0) (Finak et al., 2015) to identify genes that were differentially expressed in malignant cells compared with normal cells. Briefly, this probabilistic method takes log-transformed TPM values as input and uses the shrinkage variance estimate obtained by the empirical Bayes method. The genes with an absolute logFC > 1 and FDR < 0.05 were considered as significantly DEGs.

Then, the functional annotation and pathway enrichment analysis of genes were implemented by ClueGO (Bindea et al., 2009) with the threshold of FDR < 0.05.

### WGCNA Analysis

The co-expression network analysis was performed using Weighted Gene Co-Expression Network Analysis (WGCNA, version 1.69) (Langfelder and Horvath, 2008). The TPM values of PCGs were used as input for module detection. First, the soft threshold for network construction was selected, which was 6 here. The soft threshold made the adjacency matrix to be the continuous value between 1 and 20, so that the constructed network was conformed to be the power-law distribution and was closer to the real biological network state. Second, the scale-free network was constructed using *blockwiseModules* function, followed by the module partition analysis to identify gene co-expression modules, which could group genes with similar patterns of expression. The modules were defined by cutting the clustering tree into branches using a dynamic tree-cutting algorithm and assigned to different colors for visualization. Finally, we obtained three modules containing less than 1000 member genes. The co-expression network of each module was exported using *exportNetworkToCytoscape* function and further visualized by Cytoscape (version 3.6.0) (Shannon et al., 2003).

### The Effects of LncRNAs on Clinical Outcomes of GBM Patients

The expression profiles of 165 GBM samples from TCGA were downloaded from https://osf.io/gqrz9/ (Tatlow and Piccolo, 2016), with the clinical information for survival analysis obtained from the public cBio Cancer Genomics Portal^1^ (Cerami et al., 2012; Gao et al., 2013). The overall survival and disease-free survival were used as the end points. The Kaplan–Meier method was used for the visualization purposes and the differences between survival curves were calculated by log-rank test. The *P* values less than 0.05 were considered to be statistically significant. All of these statistical analyses were performed using R software^2^, version 3.4.4.

### Clustering of GBM Cells in Validation Data From Neftel et al. (2019)

Clustering cells was performed using Monocle (version 2.6.4) (Trapnell et al., 2014) with regressing out the patient effect. We used the *reduceDimension* function, which actually used the *lmFit* function in R package limma (Ritchie et al., 2015) to remove the patient effect on gene expression. We selected genes with average expression level more than 0.1 and high dispersion for clustering, which were marked using *setOrderingFilter* function. Then *clusterCells* function was used to cluster cells in an unsupervised manner, with parameters rho_threshold = 2 and delta_threshold = 4. Monocle employs a density-based approach (Rodriguez and Laio, 2014) to automatically cluster cells based on each cell’s local density (rho_threshold) and the nearest distance (delta_threshold) to another cell with higher distance. Certain cells with local density and distance more than the thresholds are considered as the density peaks, which are then used to identify the clusters for all cells. We finally identified 15 cell clusters in validation data from Neftel et al. (2019)

### Estimation of Activity for Diverse Signatures

The GSVA scores of EMT were calculated using predefined gene sets (Supplementary Table 1) extracted from the Molecular Signatures Database (MSigDB) (Liberzon et al., 2011). For invasive scores and cell type scores, we calculated the mean expression levels of GBM invasion-associated genes which were manually extracted from previous studies (Supplementary Table 1) and brain cell type-specific markers defined by Darmanis et al. (2015).

## Results

### The Characterization of the Dysregulated Transcriptome in GBM

**FIGURE 1:**
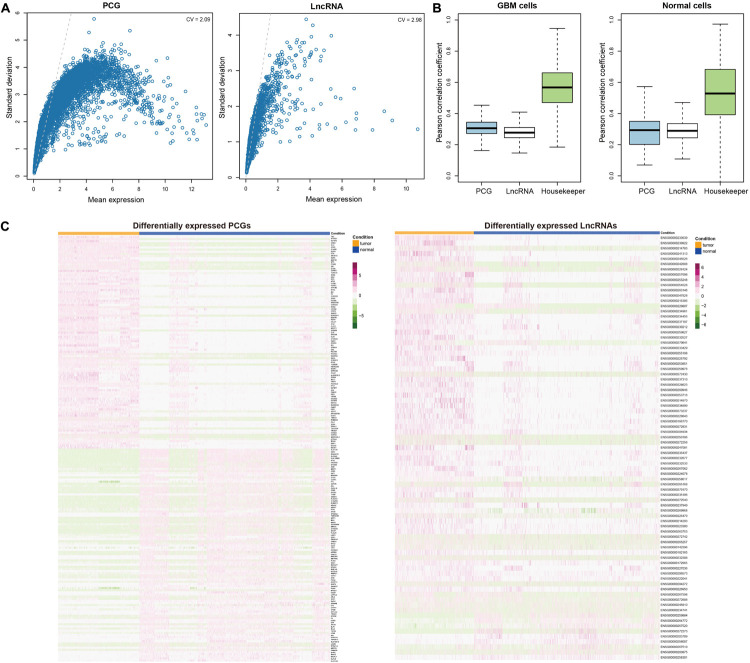
Characterization of dysregulated transcriptome in GBM at single cell level. **(A)** Scatter plots evaluating the average expression levels of PCGs (left) and lncRNAs (right) with their variations across cells, respectively. **(B)** Comparison of correlation coefficients between cells which were calculated based on the expression levels of PCGs, lncRNAs and housekeepers in GBM cells (left) and normal brain cells (right). **(C)** Heatmaps showing the top 100 upregulated PCGs and top 100 downregulated PCGs (left) and all differentially expressed lncRNAs (right). Each row represents one PCG or lncRNA and each column represents a cell. Orange denotes the GBM cells and blue denotes the normal cells.

Although previous studies have reported the close relationships of PCGs and lncRNAs with cancers using bulk RNA sequencing data (Chen Q. et al., 2018; Tao et al., 2020), few have focused on the roles of lncRNAs in tumorigenesis and progression of GBM at single-cell level. To address this issue, we initially downloaded the single-cell RNA-seq data of 3589 cells from four GBM patients [published by Darmanis et al. (2017)]. After preprocessing and quality control (see section “Materials and Methods”), we retained 998 GBM cells and 2347 normal cells with 11520 PCGs and 1877 lncRNAs. Compared with PCGs, most of lncRNAs showed relatively lower expression levels on average (Figure 1A). However, we also observed a small part of lncRNAs had comparably high expression levels with PCGs. And lncRNAs had more variable expression as shown by the high coefficient of variation (CV) for averaged expression than PCGs (CV = 2.98 for lncRNAs and CV = 2.09 for PCGs), suggesting their potential functional relevance. This was supported by the observations that the Spearman rank correlation coefficients calculated between any two cell pairs for lncRNAs were significantly lower than those for PCGs in both GBM cells and normal cells (Wilcoxon rank sum test, *P* < 0.001, Figure 1B).

To capture the functional molecules during tumorigenesis, we further utilized MAST (Finak et al., 2015), which was specifically designed for single-cell RNA-seq data to identify the DEGs between GBM and normal cells (see section “Materials and Methods”). We totally identified 2050 upregulated and 385 downregulated PCGs (Figure 1C and Supplementary Table 2), among which *TNC* (Nie et al., 2015; Xia et al., 2016), *IGFBP2* (Hsieh et al., 2010; Patil et al., 2015), and *EGFR* (Giannini et al., 2005; Beck et al., 2011) ranked in the top 10 DEGs and were all reported to be associated with gliomagenesis and GBM invasion. Functional enrichment analysis revealed that the upregulated PCGs were involved in biological processes like glial cell differentiation, glial cell proliferation and regulation of neurotransmitter transport and the downregulated PCGs mainly participated in defense response and regulation of neurons, such as myeloid leukocyte mediated immunity, regulation of leukocyte apoptotic process, cytokine production involved in immune response and negative regulation of neuron apoptotic process (Supplementary Figure 1). Moreover, we obtained 72 upregulated and 9 downregulated lncRNAs (Figure 1C and Supplementary Table 2). Besides some well-known cancer-associated lncRNAs such as *LINC01158* (Li Y. et al., 2018), *LINC00461* (Dong et al., 2019), *XIST* (Yu et al., 2017), and *HOTAIRM1* (Li Q. et al., 2018), we also identified several potential GBM progression-associated lncRNAs like *POLR2J4*, *WWTR1-AS1*, and *VIM-AS1*.

### Identification of GBM-Associated Modules at Single-Cell Level

**FIGURE 2:**
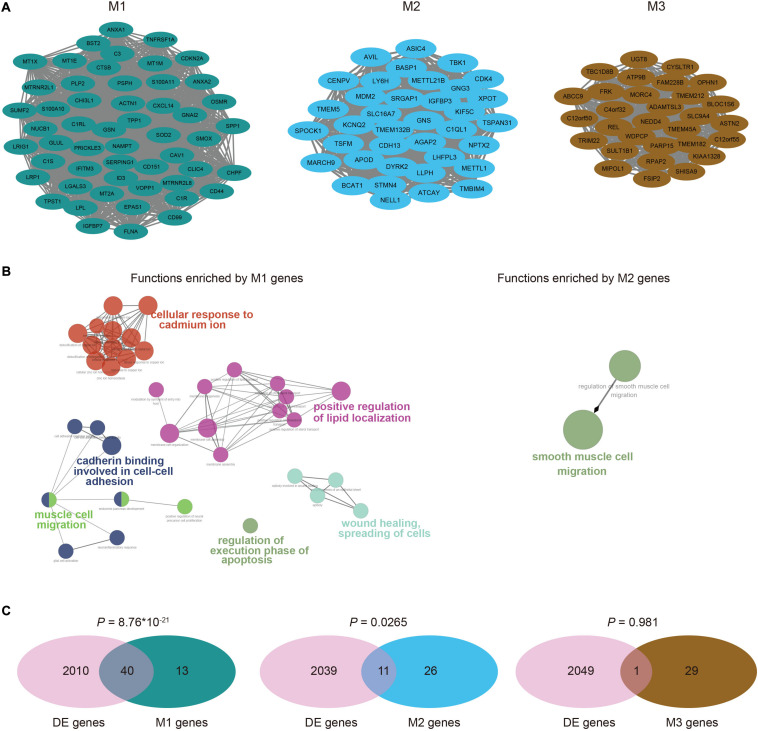
Co-expressed modules identified by WGCNA. **(A)** The co-expression network of module M1, M2, and M3, visualized by Cytoscape. **(B)** Functional annotations for genes in M1 and M2, which were implemented by ClueGO. There were no functions enriched by M3 genes. **(C)** Venn diagrams showed the significant overlaps of genes in each module with differentially expressed genes, except for M3. *P* values were calculated by hypergeometric test.

Since genes usually synergistically play important roles in tumorigenesis, we performed WGCNA (Langfelder and Horvath, 2008) on the PCG expression profiles of GBM cells to identify highly co-expressed clusters of genes (see section “Materials and Methods”). We finally obtained three modules (M1, M2, and M3), which contained 53, 37, and 30 genes, respectively. The genes in each module were highly connected to form a tight network structure (Figure 2A), showing strong correlations of expression levels with each other (Supplementary Figure 2). To determine the contribution of each module to gliomagenesis and progression, we performed the functional enrichment analysis of module genes. M1 genes were mainly involved in cell-cell adhesion, wound healing and spreading of cells, cell migration and positive regulation of lipid localization (Figure 2B). M2 genes were only enriched into one biological process of smooth muscle cell migration and there were no functions enriched by M3 genes. The pathway enrichment analysis on the genes in the three modules revealed that M1 genes were involved in human complement system, zinc homeostasis and senescence and autophagy in cancer (Supplementary Figure 3). M2 genes were only enriched into p52 signaling pathway while none pathways were enriched by M3 genes. Moreover, we found that M1 showed a significant overlap with DEGs (hypergeometric test, *P* = 8.76 × 10 ^–21^), accounting for 75.5 percentage (40/53) of module genes (Figure 2C). M2 contained 11 DEGs, which accounted for 29.7 percentage (11/37) of modules, while there was no significant overlap between M3 genes and DEGs since M3 contained only one DEG. These results implied the critical roles of these modules in the tumorigenesis and progression of GBM, especially for M1.

### Determination of GBM Invasion-Associated Module

**FIGURE 3:**
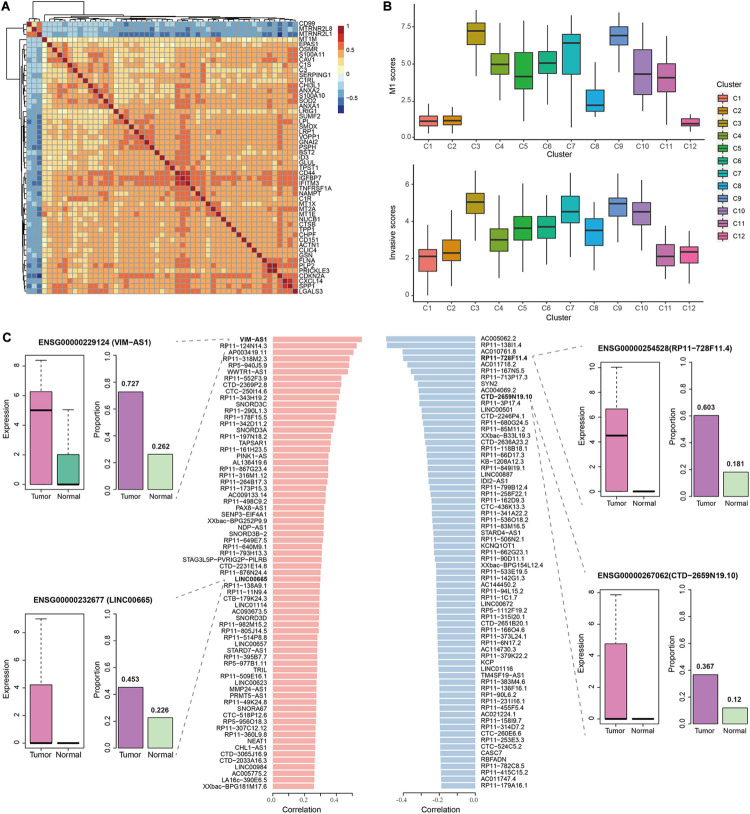
The correlation of M1 with GBM invasion. **(A)** Heatmap showing the Spearman correlation coefficients of expression levels for any gene pair in M1. **(B)** Boxplots showing the M1 scores (top) and invasive scores (bottom) of each cell cluster identified by our previous work using the same data. The GBM invasion-associated markers were manually extracted from previous studies. **(C)** Barplots in the middle showing the significant Spearman correlation coefficients of top 100 positively (left) and negatively (right) lncRNAs between their expression levels and M1 scores. In the examples of lncRNAs, boxplots represent the expression levels of the corresponding lncRNA in tumor cells and normal cells, while barplots represent the proportion of cell with their detected expression.

Since M1 was the most significant and largest module that enriched for DEGs, we further assessed its contribution to GBM progression. Most M1 genes showed relatively high positive correlations of expression levels with each other, except for *CD99*, *MTRNR2L1*, and *MTRNR2L2* (Figure 3A). Notably, many DEGs in M1 have been reported to be associated with migration and invasion. For example, EPAS1 was an important transcription factor (TF) that was validated to promote the invasive potential of GBM cells by our previous work (Pang et al., 2019). Many studies revealed that ANX family proteins (ANXA1 and ANXA2), especially ANXA2, could promote cancer progression including proliferation, invasion and metastasis (Chen C.Y. et al., 2018). The S100 proteins such as S100A11 could promote GBM progression through ANXA2-mediated NF-κB signaling pathway (Tu et al., 2019) and S100A10 could form heterotetramers with ANXA2 to promote the activation of matrix metalloproteases (MMPs) to increase the invasive ability of tumor cells (Chen C.Y. et al., 2018). Interestingly, *ANXA1*, *ANXA2*, *S100A10*, and *S100A11* were all contained in M1 and represented high correlation, especially for *ANXA2* and *S100A10*. These observations suggested the potential association of M1 with GBM invasion.

To validate the above observations, we combined the results from our previous work (Pang et al., 2019), in which we identified 12 cell clusters using the same data set. And cluster 3, 4, 7, and 9 showed relatively higher expression of EMT-associated genes. Here, we calculated the mean expression levels of M1 genes as the M1 scores in each cell of clusters and found that cluster 3 displayed the highest M1 scores, followed by cluster 7 and 9 (Figure 3B), which was consistent with our previous observations. However, we similarly calculated the M2 and M3 scores and found that cluster 5 and 10 showed higher M2 scores and cluster 4 and 11 showed higher M3 scores (Supplementary Figure 4). Further, we collected experimentally validated genes that could contribute to the invasive ability of glioblastoma cells (such as *ZEB1*, *HNRNPC*, *WNT5A*, and *DRAM1*) to evaluate the invasive scores for each cell (see section “Materials and Methods”). Similar results were observed that those three cell clusters were the top-ranked ones with high invasive scores (Figure 3B), which further supported the contribution of M1 to GBM invasion.

### Identification of GBM Invasion-Associated LncRNAs

**FIGURE 4:**
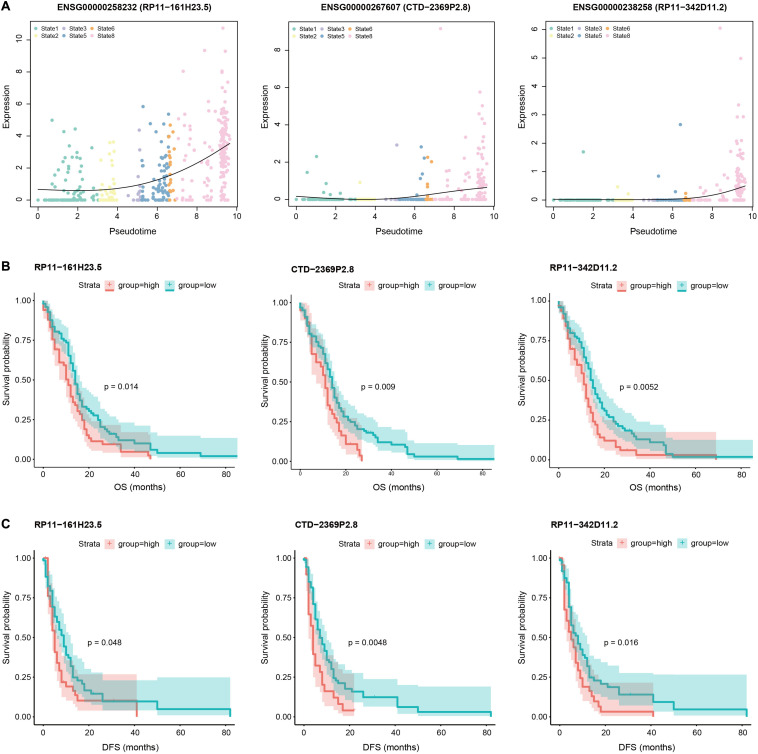
Pseudotime and survival analysis of invasion-associated lncRNAs. **(A)** Scatter plots showing the expression levels of three example lncRNAs (RP11-161H23.5, CTD-2369P2.8, and RP11-342D11.2) increase as a function of pseudotime in “stem-to-invasion” path that identified in our previous work, containing state 1, 2, 3, 5, 6, and 8 cells. A natural spline was used to model gene expression as a smooth, non-linear function over pseudotime. **(B)** Comparison of overall survival among patients with high expression levels of these three lncRNAs (red line) and those with low expression levels of corresponding lncRNAs (green line) by Kaplan–Meier analysis (with log-rank *P* values) in the cohort of 165 GBM patients. The patients were divided into two groups based on the average expression level of corresponding lncRNAs across all patients. **(C)** Comparison of disease-free survival among patients with high expression levels of these three lncRNAs (red line) and those with low expression levels of corresponding lncRNAs (green line) by Kaplan–Meier analysis (with log-rank *P* values) in the cohort of 165 GBM patients. The patients were divided into two groups based on the average expression level of corresponding lncRNAs across all patients.

Given the close association of M1 with GBM invasion, we next calculated the Spearman rank correlation coefficients between the expression levels of each lncRNA and M1 scores across all GBM cells and identified 1264 significantly correlated lncRNAs (including 611 positively correlated lncRNAs and 653 negatively correlated lncRNAs, Supplementary Table 3), which were considered as GBM invasion-associated lncRNAs. The top 100 positively and negatively correlated lncRNAs were shown in Figure 3C. For example, among the positively correlated lncRNAs, *VIM-AS1* ranked among the top one with the correlation coefficient of 0.56, which was upregulated in GBM cells with a higher expressed proportion (72.7%) compared to normal cells (26.2%). Previous studies also revealed that the high expression of *VIM-AS1* was positively associated with patients’ worse prognosis (Suo et al., 2020). Other lncRNAs like *WWTR1-AS1* and *LINC00665* similarly showed significantly higher expression levels and cell proportions in tumor cells. For negatively correlated lncRNAs, *ENSG00000254528* (*RP11-728F11.4*) and *ENSG00000267062* (*CTD-2659N19.10*) ranked among the top four and ten, both of which showed significantly higher expression levels in GBM cells and nearly no expression in normal cells. Notably, *VIM-AS1* and *WWTR1-AS1* were the top two lncRNAs with the highest correlations between their expression levels and pseudotime along the “stem-to-invasion path” in our previous work (Pang et al., 2019). These findings promoted us to explore the dynamic changes of GBM invasion-associated lncRNAs along the “stem-to-invasion path.” We found that the expression levels of many lncRNAs such as *ENSG00000258232* (*RP11-161H23.5*), *ENSG00000267607* (*CTD-2369P2.8*), and *ENSG00000238258* (*RP11-342D11.2*), gradually increased as cells transferred from cancer stem cell-like state to invasive state (Figure 4A). These consistent results confirmed the potential roles of these lncRNAs on GBM invasion.

Given that cancer-associated mortality is principally attributable to the development of invasion and metastasis, we speculated that these GBM invasion-associated lncRNAs might be of importance in determining patient outcomes. Next, we performed survival analysis using the expression profiles and clinical information of 165 GBM patients (see section “Materials and Methods”). Among invasion-associated lncRNAs, several of them showed significant correlations with prognosis of patients. For example, the overall survival (OS) of patients with high expression levels of *ENSG00000258232* (*RP11-161H23.5*), *ENSG00000267607* (*CTD-2369P2.8*), and *ENSG00000238258* (*RP11-342D11.2*) were significantly shorter than those with low expression levels (*P* = 0.014, *P* = 0.009, and *P* = 0.0052, respectively, Figure 4B). Moreover, patients with high expression levels of these three lncRNAs also had worse disease-free survival (DFS) than those with low expression levels (*P* = 0.048, *P* = 0.0048, and *P* = 0.016, respectively, Figure 4C). These results suggested potential implication of invasion-associated genes in GBM tumorigenesis, progression and prognosis.

### Validation of the Invasion-Associated Module and LncRNAs by Extra Data of GBM

To validate the contribution of M1 genes and lncRNAs to GBM invasion, we downloaded another single-cell RNA-seq data of 28 GBM patients [published by Neftel et al. (2019)]. After quality control, we retained 6863 GBM cells and 1067 normal cells with 11441 PCGs and 585 lncRNAs, in which the numbers of commonly detected PCGs and lncRNAs in both data sets were 11441 and 192, respectively. In this validation data, we identified 1676 DEGs and 13 dysregulated lncRNAs (Supplementary Table 4), among which 1066 DEGs and 6 dysregulated lncRNAs were shared by both data sets.

**FIGURE 5:**
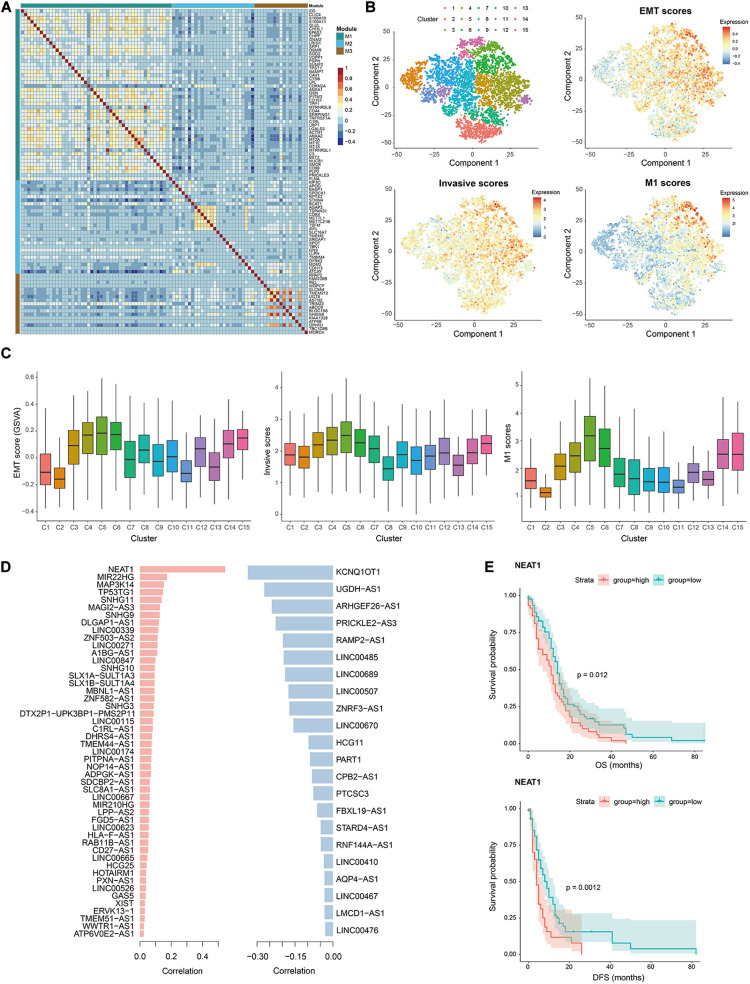
Validation of invasion-associated M1 and lncRNAs using data from Neftel et al. (2019) **(A)** Heatmap showing the Spearman correlation coefficients of expression levels for any gene pair in M1, M2, and M3. **(B)** T-SNE plots of tumor cells showing 15 clusters and the EMT scores, invasive scores and M1 scores in each cell. Red denotes high scores and blue denote low scores. **(C)** Comparison of EMT scores, invasive scores and M1 scores in cells of each cluster, indicating the similar distribution as cluster 5, 6, 14, and 15 display relatively higher scores. **(D)** List of commonly identified positively (left) and negatively (right) lncRNAs as well as their Spearman correlation coefficient with M1 scores in this validation data. **(E)** Comparison of overall (top) and disease-free (bottom) survival among patients with high expression levels (red line) of lncRNA NEAT1 and those with low expression levels (green line) by Kaplan–Meier analysis (with log-rank *P* values) in the cohort of 165 GBM patients. The patients were divided into two groups based on the average expression level of NEAT1 across all patients.

We recaptured the modularity of M1 genes in this validation data as they showed stronger co-expression pattern compared to the other two module genes (Figure 5A), suggesting their functional synergy. The similar patterns were observed in data from children and adults with GBM, respectively (Supplementary Figure 5). To determine whether M1 genes were involved in GBM invasion, we first used Monocle (Trapnell et al., 2014) to group GBM cells into 15 clusters, excluding patient-specific effects with linear regression (see section “Materials and Methods,” Figure 5B and Supplementary Figure 6). Each cluster consisted of cells from multiple patients (Supplementary Figure 7). Then we calculated the EMT, invasive and M1 scores as above for each cell and found that they showed quite similar distribution patterns (Figures 5B,C). Cluster 5 and 6 consistently had the highest scores, followed by cluster 4, 14, and 15, which located adjacent to each other in the transcriptome space of Figure 5B. These results again confirmed the association of M1 genes with GBM invasion.

Therefore, we calculated the Spearman rank correlation coefficients between the expression levels of each invasion-associated lncRNA identified in data from Darmanis et al. This resulted in 71 significantly correlated lncRNAs (including 49 positively correlated lncRNAs and 22 negatively correlated lncRNAs, Supplementary Table 5) among the 192 commonly detected lncRNAs. Notably, NEAT1 was the top one lncRNA with a positive correlation coefficient of 0.54 in validation data (Figure 5D), which also ranked among the top 63 in the data from Darmanis et al. Moreover, the high expression level of NEAT1 was significantly correlated with poor OS and DFS of patients (Figure 5E), which was accordant with the roles of NEAT1 in promoting malignant phenotypes and progression of GBM (Chen Q. et al., 2018; Zhou et al., 2019). All these results again validated the contributions of the identified lncRNAs to GBM invasion and progression.

## Discussion

The fast and invasive growth is the hallmark of GBM, which is a major factor contributing to dismal outcomes (Du et al., 2008). Therefore, understanding the molecular mechanisms underlying tumor cell invasion and migration is crucial for the treatment of GBM. Although previous studies have made massive efforts to identify many PCGs and lncRNAs promoting glioblastoma cell invasion using bulk sequencing data, few have actually achieved successful clinical application. In this study, we utilized single-cell RNA-seq data from multiple GBM patients to dissect invasion-associated factors including lncRNAs, which provided new insights into the development and progression of glioblastoma.

Central to our understanding of glioblastoma biology is the idea that a subpopulation of glioblastoma stem cells drives tumorigenesis and progression (Singh et al., 2004). Lan et al. (2017) analyzed the growth dynamics of GBM clones and revealed that the initiation of human GBM may result from the aberrant reactivation of a normal developmental program. Couturier et al. (2020) compared the lineage hierarchy of the developing human brain to the transcriptome of 53586 adult glioblastoma cells at single-cell level and found that glioblastoma development recapitulates a normal neurodevelopmental hierarchy. These findings suggested the important roles of the development system in tumorigenesis and progression of GBM and were also supported by many other studies (Filbin et al., 2018; Yuan et al., 2018). Consistently, in this work, we identified dysregulated PCGs and lncRNAs and the functional enrichment analyses showed that these PCGs participated in brain development-associated biological processed, such as glial cell differentiation and glial cell proliferation. This implied that we indeed captured the potential key factors contributing to GBM initiation and progression.

Since invasion and metastasis are the late events during the course of multi-step tumor progression (Lambert et al., 2017), which result in the vast majority of deaths from cancer (Coghlin and Murray, 2010), we seek to identify critical factors, especially lncRNAs, that are involved in the regulation of GBM invasion. Given the lack of functional annotation of lncRNAs, we first identified co-expressed PCG modules by WGCNA to determine the invasion-associated genes. Among the three modules, M1 significantly enriched the largest number of differentially expressed PCGs, many of which have been reported the association with GBM invasion, such as *EPAS1*, *ANXA2* and its target gene *OSMR* (Matsumoto et al., 2020). And ANAX2 was also the target of lncRNA LINC00941, which was one of the invasion-assocaited lncRNAs. Previous studies have revealed that S100A10 could form a heterotetramer with ANXA2 to promote tumor cell invasion (Chen C.Y. et al., 2018) and S100A11 could also interact with ANXA1 which is a Ca 2^+^-regulated phospholipid-binding protein (Boudhraa et al., 2016) to form Ca 2^+^-dependent heterotetramers. These genes were all contained in M1 with high expression in GBM cells, underlying the functions of cellular response to cadmium ion (Figure 2B) enriched by M1 genes, which might be a potential molecular mechanism of GBM invasion. Surprisingly, although most of M1 genes showed positive correlations, *CD99*, *MTRNR2L1*, and *MTRNR2L8* were negatively correlated with others. As it has been widely reported that overexpression of *CD99* could increase the migration and invasiveness of GBM cells (Seol et al., 2012; Cardoso et al., 2019), we deduced that although *CD99* and other invasion-associated PCGs play key roles in regulating tumor cell invasion, their mediated mechanisms were distinct and redundant, resulting in their mutually exclusive expression patterns. Moreover, combining our previous work for characterization of cell clusters and construction of progression trajectory, we further confirmed the contribution of M1 to GBM invasion as M1 genes showed relatively high expression in cell clusters with high EMT and invasive scores. Interestingly, we calculated the average expression levels of cell type-specific markers defined by previous study (Darmanis et al., 2015) as the cell type scores in each cluster and found that cluster 3, 4, 7, and 9 with higher M1 scores consistently showed the highest expression levels of microglia cell markers (Supplementary Figure 8), implying the roles of microglia in GBM invasion. These observations were also recaptured in another single-cell RNA-seq data of GBM, suggesting the accuracy and repeatability of our findings.

Based on the determination of the invasion-associated module, we further identified the invasion-associated lncRNAs. In data from Darmanis et al., we found that *VIM-AS1* and *WWTR1-AS1* ranked among the top 1 and 6 in positively correlated lncRNAs with higher expression in GBM cell compared to normal cells. Notably, their expression gradually increased along the “stem-to-invasion path” in our previous work (Pang et al., 2019), confirming their roles in GBM invasion. In validation data from Neftel et al. (2019) *NEAT1* was the top one positively correlated lncRNA and MIAT was the top one negatively correlated lncRNA, consistent with their roles in GBM progression that increased *NEAT1* could promote proliferation, malignant phenotypes and TMZ resistance (Bi et al., 2020) and high expression of *MIAT* is associated with prolonged survival (Bountali et al., 2019). However, we did not recapture the top-ranked lncRNAs like *VIM-AS1* and *WWTR1-AS1* as they were not detected in validation data. This may result from the generally lower expression levels of lncRNAs compared to PCGs and the inherent limitations of scRNA-seq like high dropout rates and data sparsity. Actually, among the 192 commonly detected lncRNAs, 71 were consistently identified as invasion-associated lncRNAs in both data sets, indicating the robustness of our results.

In summary, our work took advantage of scRNA-seq to identify and dissect the GBM invasion-associated lncRNAs and their effect on clinical outcomes at a high resolution, providing new insights into the molecular mechanism of the development and progression of GBM and new potential targets for the treatment of invasive glioblastoma and possibly other solid malignant tumors.

## Data Availability Statement

Publicly available datasets were analyzed in this study. This data can be found here: GEO database (GSE84465 and GSE131928).

## Author Contributions

LP and YL designed the research. FQ, YP, and JH collected and preprocessed the data. BP, FQ, and LP performed the bioinformatics analysis. BP and LP wrote the manuscript. All authors read and approved the final manuscript.

## Conflict of Interest

The authors declare that the research was conducted in the absence of any commercial or financial relationships that could be construed as a potential conflict of interest.

## Footnotes

1 http://www.cbioportal.org

## References

[B1] Batista P. J. Chang H. Y. (2013). Long noncoding RNAs: cellular address codes in development and disease. *Cell* 152 1298–1307. 10.1016/j.cell.2013.02.012 23498938 PMC3651923

[B2] Beck S. Jin X. Sohn Y. W. Kim J. K. Kim S. H. Yin J. (2011). Telomerase activity-independent function of TERT allows glioma cells to attain cancer stem cell characteristics by inducing EGFR expression. *Mol. cells* 31 9–15. 10.1007/s10059-011-0008-8 21193962 PMC3906874

[B3] Bi C. L. Liu J. F. Zhang M. Y. Lan S. Yang Z. Y. Fang J. S. (2020). LncRNA NEAT1 promotes malignant phenotypes and TMZ resistance in glioblastoma stem cells by regulating let-7g-5p/MAP3K1 axis. *Biosci. Rep.* 40 BSR20201111. 10.1042/BSR20201111 PMC7601351 33057597

[B4] Bindea G. Mlecnik B. Hackl H. Charoentong P. Tosolini M. Kirilovsky A. (2009). ClueGO: a cytoscape plug-in to decipher functionally grouped gene ontology and pathway annotation networks. *Bioinformatics* 25 1091–1093. 10.1093/bioinformatics/btp101 19237447 PMC2666812

[B5] Boudhraa Z. Bouchon B. Viallard C. D’Incan M. Degoul F. (2016). Annexin A1 localization and its relevance to cancer. *Clin. Sci.* 130 205–220. 10.1042/cs20150415 26769657

[B6] Bountali A. Tonge D. P. Mourtada-Maarabouni M. (2019). RNA sequencing reveals a key role for the long non-coding RNA MIAT in regulating neuroblastoma and glioblastoma cell fate. *Int. J. Biol. Macromol.* 130 878–891. 10.1016/j.ijbiomac.2019.03.005 30836187

[B7] Cardoso L. C. Soares R. D. S. Laurentino T. S. Lerario A. M. Marie S. K. N. Oba-Shinjo S. M. (2019). CD99 expression in glioblastoma molecular subtypes and role in migration and invasion. *Int. J. Mol. Sci.* 20:1137. 10.3390/ijms20051137 30845661 PMC6429353

[B8] Cerami E. Gao J. Dogrusoz U. Gross B. E. Sumer S. O. Aksoy B. A. (2012). The cBio cancer genomics portal: an open platform for exploring multidimensional cancer genomics data. *Cancer Discov.* 2 401–404. 10.1158/2159-8290.cd-12-0095 22588877 PMC3956037

[B9] Chen C. Y. Lin Y. S. Chen C. H. Chen Y. J. (2018). Annexin A2-mediated cancer progression and therapeutic resistance in nasopharyngeal carcinoma. *J. Biomed. Sci.* 25:30. 10.1186/s12929-018-0430-8 PMC5877395 29598816

[B10] Chen Q. Cai J. Wang Q. Wang Y. Liu M. Yang J. (2018). Long noncoding RNA NEAT1, regulated by the EGFR pathway, contributes to glioblastoma progression through the WNT/beta-catenin pathway by scaffolding EZH2. *Clin. Cancer Res.* 24 684–695. 10.1158/1078-0432.ccr-17-0605 29138341

[B11] Chung W. Eum H. H. Lee H. O. Lee K. M. Lee H. B. Kim K. T. (2017). Single-cell RNA-seq enables comprehensive tumour and immune cell profiling in primary breast cancer. *Nat. Commun.* 8:15081. 10.1038/ncomms15081 PMC5424158 28474673

[B12] Coghlin C. Murray G. I. (2010). Current and emerging concepts in tumour metastasis. *J. Pathol.* 222 1–15. 10.1002/path.2727 20681009

[B13] Couturier C. P. Ayyadhury S. Le P. U. Nadaf J. Monlong J. Riva G. (2020). Single-cell RNA-seq reveals that glioblastoma recapitulates a normal neurodevelopmental hierarchy. *Nat. Commun.* 11 3406. 10.1038/s41467-020-17186-5 PMC7343844 32641768

[B14] Darmanis S. Sloan S. A. Croote D. Mignardi M. Chernikova S. Samghababi P. (2017). Single-Cell RNA-Seq analysis of infiltrating neoplastic cells at the migrating front of human glioblastoma. *Cell Rep.* 21 1399–1410. 10.1016/j.celrep.2017.10.030 29091775 PMC5810554

[B15] Darmanis S. Sloan S. A. Zhang Y. Enge M. Caneda C. Shuer L. M. (2015). A survey of human brain transcriptome diversity at the single cell level. *Proc. Natl. Acad. Sci. U.S.A.* 112 7285–7290. 10.1073/pnas.1507125112 26060301 PMC4466750

[B16] Dong L. Qian J. Chen F. Fan Y. Long J. (2019). LINC00461 promotes cell migration and invasion in breast cancer through miR-30a-5p/integrin beta3 axis. *J. Cell. Biochem.* 120 4851–4862. 10.1002/jcb.27435 30623482

[B17] Du R. Petritsch C. Lu K. Liu P. Haller A. Ganss R. (2008). Matrix metalloproteinase-2 regulates vascular patterning and growth affecting tumor cell survival and invasion in GBM. *Neuro Oncol.* 10 254–264. 10.1215/15228517-2008-001 18359864 PMC2563048

[B18] Filbin M. G. Tirosh I. Hovestadt V. Shaw M. L. Escalante L. E. Mathewson N. D. (2018). Developmental and oncogenic programs in H3K27M gliomas dissected by single-cell RNA-seq. *Science* 360 331–335. 29674595 10.1126/science.aao4750 PMC5949869

[B19] Finak G. McDavid A. Yajima M. Deng J. Gersuk V. Shalek A. K. (2015). MAST: a flexible statistical framework for assessing transcriptional changes and characterizing heterogeneity in single-cell RNA sequencing data. *Genome Biol.* 16:278. 10.1186/s13059-015-0844-5 PMC4676162 26653891

[B20] Gao J. Aksoy B. A. Dogrusoz U. Dresdner G. Gross B. Sumer S. O. (2013). Integrative analysis of complex cancer genomics and clinical profiles using the cBioPortal. *Sci. Signal.* 6:l1. 10.1126/scisignal.2004088 PMC4160307 23550210

[B21] Giannini C. Sarkaria J. N. Saito A. Uhm J. H. Galanis E. Carlson B. L. (2005). Patient tumor EGFR and PDGFRA gene amplifications retained in an invasive intracranial xenograft model of glioblastoma multiforme. *Neuro Oncol.* 7 164–176. 10.1215/s1152851704000821 15831234 PMC1871885

[B22] Hanniford D. Ulloa-Morales A. Karz A. Berzoti-Coelho M. G. Moubarak R. S. Sanchez-Sendra B. (2020). Epigenetic silencing of CDR1as drives IGF2BP3-mediated melanoma invasion and metastasis. *Cancer cell* 37 55–70 e15. 31935372 10.1016/j.ccell.2019.12.007 PMC7184928

[B23] Hovestadt V. Smith K. S. Bihannic L. Filbin M. G. Shaw M. L. Baumgartner A. (2019). Resolving medulloblastoma cellular architecture by single-cell genomics. *Nature.* 572 74–79. 31341285 10.1038/s41586-019-1434-6 PMC6754173

[B24] Hsieh D. Hsieh A. Stea B. Ellsworth R. (2010). IGFBP2 promotes glioma tumor stem cell expansion and survival. *Biochem. Biophys. Res. Commun.* 397 367–372. 10.1016/j.bbrc.2010.05.145 20515648

[B25] Lambert A. W. Pattabiraman D. R. Weinberg R. A. (2017). Emerging biological principles of metastasis. *Cell* 168 670–691. 10.1016/j.cell.2016.11.037 28187288 PMC5308465

[B26] Lan X. Jorg D. J. Cavalli F. M. G. Richards L. M. Nguyen L. V. Vanner R. J. (2017). Fate mapping of human glioblastoma reveals an invariant stem cell hierarchy. *Nature* 549 227–232. 28854171 10.1038/nature23666 PMC5608080

[B27] Langfelder P. Horvath S. (2008). WGCNA: an R package for weighted correlation network analysis. *BMC Bioinformatics* 9:559. 10.1186/1471-2105-9-559 19114008 PMC2631488

[B28] Langmead B. Trapnell C. Pop M. Salzberg S. L. (2009). Ultrafast and memory-efficient alignment of short DNA sequences to the human genome. *Genome Biol.* 10:R25. 10.1186/gb-2009-10-3-r25 PMC2690996 19261174

[B29] Li B. Dewey C. N. (2011). RSEM: accurate transcript quantification from RNA-Seq data with or without a reference genome. *BMC Bioinformatics* 12:323. 10.1186/1471-2105-12-323 21816040 PMC3163565

[B30] Li H. Courtois E. T. Sengupta D. Tan Y. Chen K. H. Goh J. J. L. (2017). Reference component analysis of single-cell transcriptomes elucidates cellular heterogeneity in human colorectal tumors. *Nat. Genet.* 49 708–718. 10.1038/ng.3818 28319088

[B31] Li Q. Dong C. Cui J. Wang Y. Hong X. (2018). Over-expressed lncRNA HOTAIRM1 promotes tumor growth and invasion through up-regulating HOXA1 and sequestering G9a/EZH2/Dnmts away from the HOXA1 gene in glioblastoma multiforme. *J. Exp. Clin. Cancer Res.* 37:265. 10.1186/s13046-018-0941-x PMC6208043 30376874

[B32] Li Y. Li Y. Wang D. Meng Q. (2018). Linc-POU3F3 is overexpressed in hepatocellular carcinoma and regulates cell proliferation, migration and invasion. *Biomed. Pharmacother.* 105 683–689. 10.1016/j.biopha.2018.06.006 29906746

[B33] Liberzon A. Subramanian A. Pinchback R. Thorvaldsdottir H. Tamayo P. Mesirov J. P. (2011). Molecular signatures database (MSigDB) 3.0. *Bioinformatics* 27 1739–1740. 10.1093/bioinformatics/btr260 21546393 PMC3106198

[B34] Matsumoto Y. Ichikawa T. Kurozumi K. Otani Y. Fujimura A. Fujii K. (2020). Annexin A2-STAT3-Oncostatin M receptor axis drives phenotypic and mesenchymal changes in glioblastoma. *Acta Neuropathol. Commun.* 8:42. 10.1186/s40478-020-00916-7 PMC7132881 32248843

[B35] Neftel C. Laffy J. Filbin M. G. Hara T. Shore M. E. Rahme G. J. (2019). An Integrative Model of Cellular States, Plasticity, and Genetics for Glioblastoma. *Cell* 178 835–49 e21. 31327527 10.1016/j.cell.2019.06.024 PMC6703186

[B36] Nie S. Gurrea M. Zhu J. Thakolwiboon S. Heth J. A. Muraszko K. M. (2015). Tenascin-C: a novel candidate marker for cancer stem cells in glioblastoma identified by tissue microarrays. *J. Proteome Res.* 14 814–822. 10.1021/pr5008653 25469866 PMC4320683

[B37] Ostrom Q. T. Gittleman H. Fulop J. Liu M. Blanda R. Kromer C. (2015). CBTRUS statistical report: primary brain and central nervous system tumors diagnosed in the United States in 2008-2012. *Neuro Oncol.* 17Suppl. 4(Suppl. 4) iv1–iv62. 10.1093/neuonc/nov189 PMC4623240 26511214

[B38] Pang B. Xu J. Hu J. Guo F. Wan L. Cheng M. (2019). Single-cell RNA-seq reveals the invasive trajectory and molecular cascades underlying glioblastoma progression. *Mol. Oncol.* 13 2588–2603. 10.1002/1878-0261.12569 31487431 PMC6887585

[B39] Patil S. S. Railkar R. Swain M. Atreya H. S. Dighe R. R. Kondaiah P. (2015). Novel anti IGFBP2 single chain variable fragment inhibits glioma cell migration and invasion. *J. Neuro Oncol.* 123 225–235. 10.1007/s11060-015-1800-7 25944386

[B40] Prensner J. R. Iyer M. K. Sahu A. Asangani I. A. Cao Q. Patel L. (2013). The long noncoding RNA SChLAP1 promotes aggressive prostate cancer and antagonizes the SWI/SNF complex. *Nat. Genet.* 45 1392–1398. 10.1038/ng.2771 24076601 PMC3812362

[B41] Ritchie M. E. Phipson B. Wu D. Hu Y. Law C. W. Shi W. (2015). limma powers differential expression analyses for RNA-sequencing and microarray studies. *Nucleic Acids Res.* 43:e47. 10.1093/nar/gkv007 25605792 PMC4402510

[B42] Rodriguez A. Laio A. (2014). Machine learning. Clustering by fast search and find of density peaks. *Science* 344 1492–1496. 10.1126/science.1242072 24970081

[B43] Seol H. J. Chang J. H. Yamamoto J. Romagnuolo R. Suh Y. Weeks A. (2012). Overexpression of cd99 increases the migration and invasiveness of human malignant glioma cells. *Genes Cancer* 3 535–549. 10.1177/1947601912473603 23486730 PMC3591096

[B44] Shannon P. Markiel A. Ozier O. Baliga N. S. Wang J. T. Ramage D. (2003). Cytoscape: a software environment for integrated models of biomolecular interaction networks. *Genome Res.* 13 2498–2504. 10.1101/gr.1239303 14597658 PMC403769

[B45] Singh S. K. Hawkins C. Clarke I. D. Squire J. A. Bayani J. Hide T. (2004). Identification of human brain tumour initiating cells. *Nature* 432 396–401. 15549107 10.1038/nature03128

[B46] Stupp R. Mason W. P. van den Bent M. J. Weller M. Fisher B. Taphoorn M. J. (2005). Radiotherapy plus concomitant and adjuvant temozolomide for glioblastoma. *N. Engl. J. Med.* 352 987–996. 15758009 10.1056/NEJMoa043330

[B47] Suo S. T. Gong P. Peng X. J. Niu D. Guo Y. T. (2020). Knockdown of long non-coding RNA VIM-AS1 inhibits glioma cell proliferation and migration, and increases the cell apoptosis via modulation of WEE1 targeted by miR-105-5p. *Eur. Rev. Med. Pharmacol. Sci.* 24 6834–6847. 32633376 10.26355/eurrev_202006_21673

[B48] Tang F. Barbacioru C. Wang Y. Nordman E. Lee C. Xu N. (2009). mRNA-Seq whole-transcriptome analysis of a single cell. *Nat. Methods* 6 377–382. 10.1038/nmeth.1315 19349980

[B49] Tao W. Zhang A. Zhai K. Huang Z. Huang H. Zhou W. (2020). SATB2 drives glioblastoma growth by recruiting CBP to promote FOXM1 expression in glioma stem cells. *EMBO Mol. Med.* 12:e12291. 10.15252/emmm.202012291 PMC7721366 33124191

[B50] Tatlow P. J. Piccolo S. R. (2016). A cloud-based workflow to quantify transcript-expression levels in public cancer compendia. *Sci. Rep.* 6:39259. 10.1038/srep39259 PMC5159871 27982081

[B51] Thakkar J. P. Dolecek T. A. Horbinski C. Ostrom Q. T. Lightner D. D. Barnholtz-Sloan J. S. (2014). Epidemiologic and molecular prognostic review of glioblastoma. *Cancer Epidemiol. Biomarkers Prev.* 23 1985–1996. 10.1158/1055-9965.epi-14-0275 25053711 PMC4185005

[B52] Trapnell C. Cacchiarelli D. Grimsby J. Pokharel P. Li S. Morse M. (2014). The dynamics and regulators of cell fate decisions are revealed by pseudotemporal ordering of single cells. *Nat. Biotechnol.* 32 381–386. 10.1038/nbt.2859 24658644 PMC4122333

[B53] Tu Y. Xie P. Du X. Fan L. Bao Z. Sun G. (2019). S100A11 functions as novel oncogene in glioblastoma via S100A11/ANXA2/NF-kappaB positive feedback loop. *J. Cell. Mol. Med.* 23 6907–6918. 10.1111/jcmm.14574 31430050 PMC6787445

[B54] Wang G. Lunardi A. Zhang J. Chen Z. Ala U. Webster K. A. (2013). Zbtb7a suppresses prostate cancer through repression of a Sox9-dependent pathway for cellular senescence bypass and tumor invasion. *Nat. Genet.* 45 739–746. 10.1038/ng.2654 23727861 PMC4036521

[B55] Wolf K. J. Chen J. Coombes J. Aghi M. K. Kumar S. (2019). Dissecting and rebuilding the glioblastoma microenvironment with engineered materials. *Nat. Rev. Mater.* 4 651–668. 10.1038/s41578-019-0135-y 32647587 PMC7347297

[B56] Xia S. Lal B. Tung B. Wang S. Goodwin C. R. Laterra J. (2016). Tumor microenvironment tenascin-C promotes glioblastoma invasion and negatively regulates tumor proliferation. *Neuro Oncol.* 18 507–517. 10.1093/neuonc/nov171 26320116 PMC4799677

[B57] Yang D. Sun B. Dai H. Li W. Shi L. Zhang P. (2019). T cells expressing NKG2D chimeric antigen receptors efficiently eliminate glioblastoma and cancer stem cells. *J. Immunother. Cancer* 7:171. 10.1186/s40425-019-0642-9 PMC6617951 31288857

[B58] Yu H. Xue Y. Wang P. Liu X. Ma J. Zheng J. (2017). Knockdown of long non-coding RNA XIST increases blood-tumor barrier permeability and inhibits glioma angiogenesis by targeting miR-137. *Oncogenesis* 6:e303. 10.1038/oncsis.2017.7 28287613 PMC5533948

[B59] Yuan J. Levitin H. M. Frattini V. Bush E. C. Boyett D. M. Samanamud J. (2018). Single-cell transcriptome analysis of lineage diversity in high-grade glioma. *Genome Med.* 10:57. 10.1186/s13073-018-0567-9 PMC6058390 30041684

[B60] Yuan J. H. Yang F. Wang F. Ma J. Z. Guo Y. J. Tao Q. F. (2014). A long noncoding RNA activated by TGF-beta promotes the invasion-metastasis cascade in hepatocellular carcinoma. *Cancer cell* 25 666–681. 10.1016/j.ccr.2014.03.010 24768205

[B61] Zhou X. Li X. Yu L. Wang R. Hua D. Shi C. (2019). The RNA-binding protein SRSF1 is a key cell cycle regulator via stabilizing NEAT1 in glioma. *Int. J. Biochem. Cell Biol.* 113 75–86. 10.1016/j.biocel.2019.06.003 31200124

