# Supplementary material for: Dissecting the Invasion-Associated Long Non-coding RNAs Using Single-Cell RNA-Seq Data of Glioblastoma
Source: Front Genet. 2021 Jan 11;11:633455. doi: 10.3389/fgene.2020.633455 (PMC7831882; doi:10.3389/fgene.2020.633455)
Supplement: Supplementary file 6 [file Data_Sheet_1.PDF]

Supplementary Figures

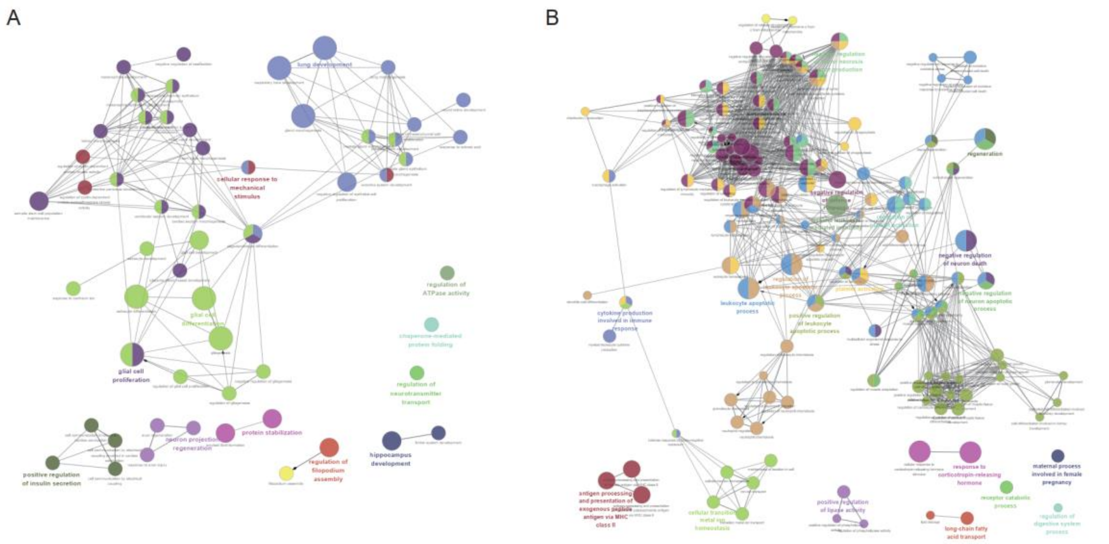

Supplementary Figure 1. The functional enrichment analysis of differentially expressed genes using ClueGO. (A) The GO terms enriched by upregulated genes. **(B)** The GO terms enriched by downregulated genes.

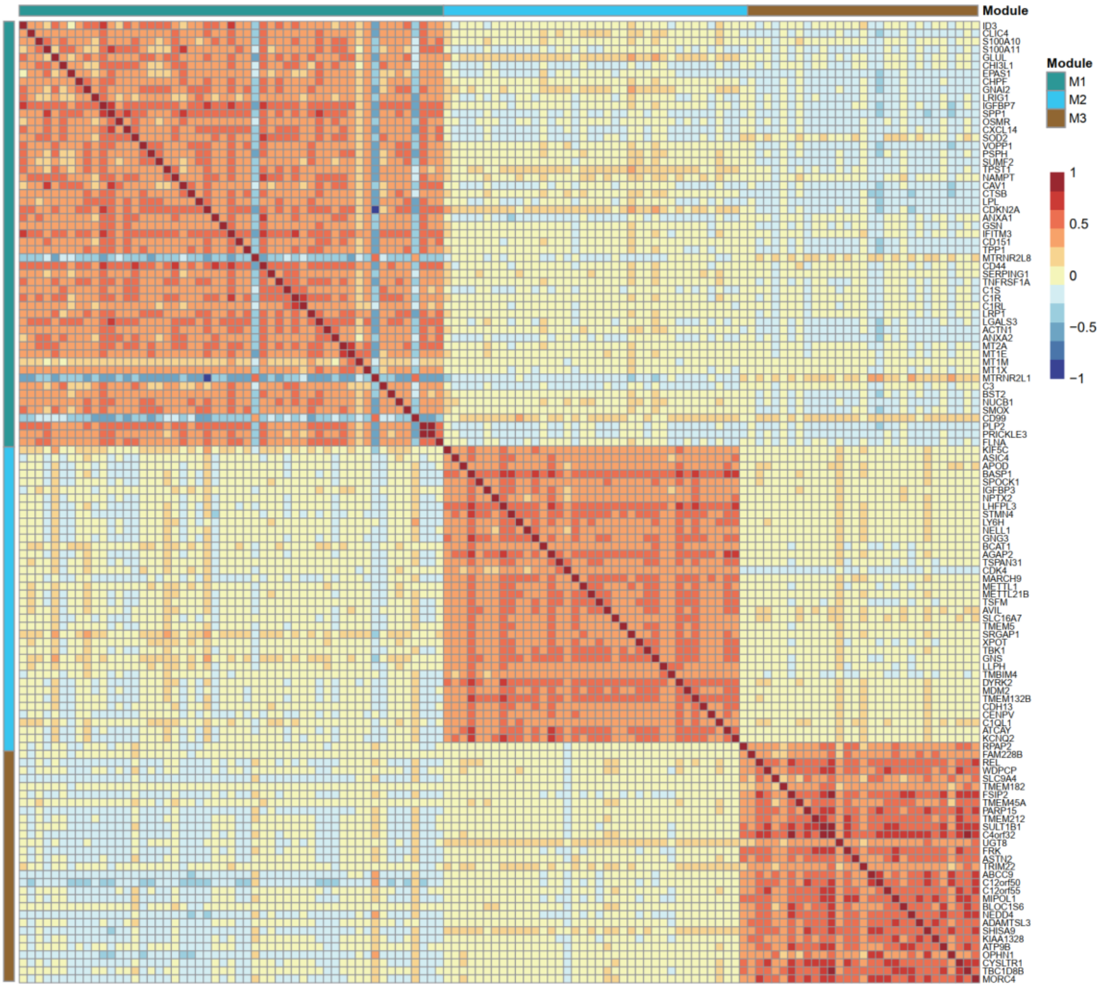

Supplementary Figure 2. The Spearman correlation coefficients of expression levels for any gene pair in M1, M2 and M3 using data from Darmanis et al.

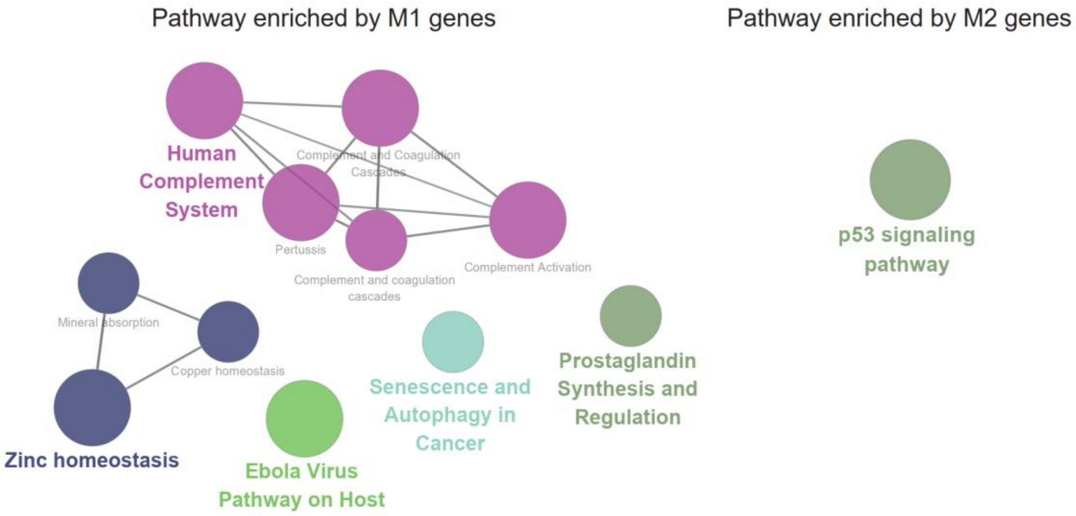

Supplementary Figure 3. The pathway enrichment analysis of M1 (left) and M2 (right) genes.

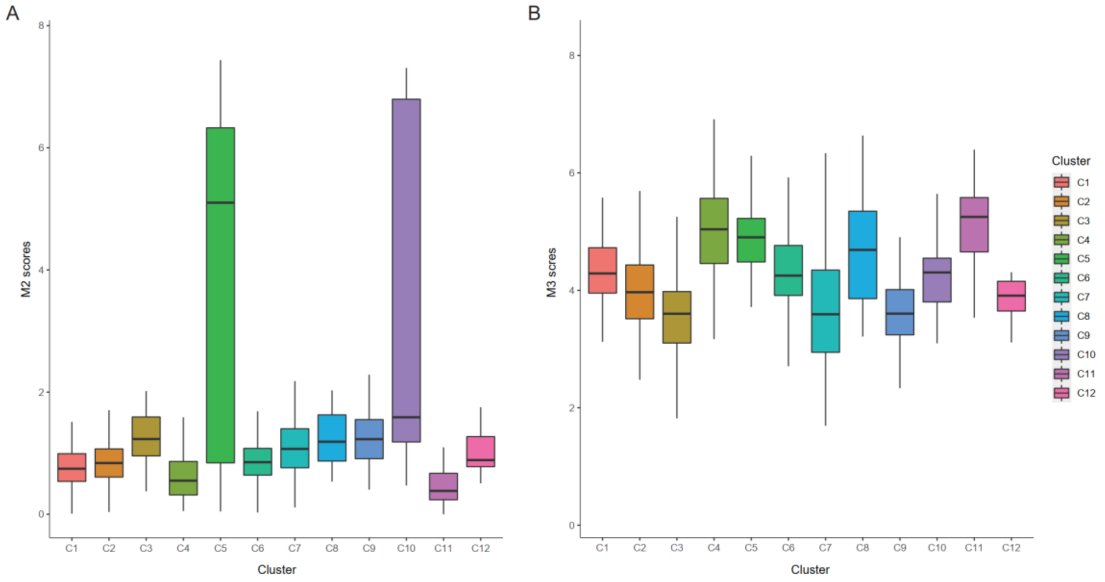

Supplementary Figure 4. The scores in each cell cluster. Boxplots showing the M2 (left) and M3 (right) scores of each cell cluster identified by our previous work using the same data.

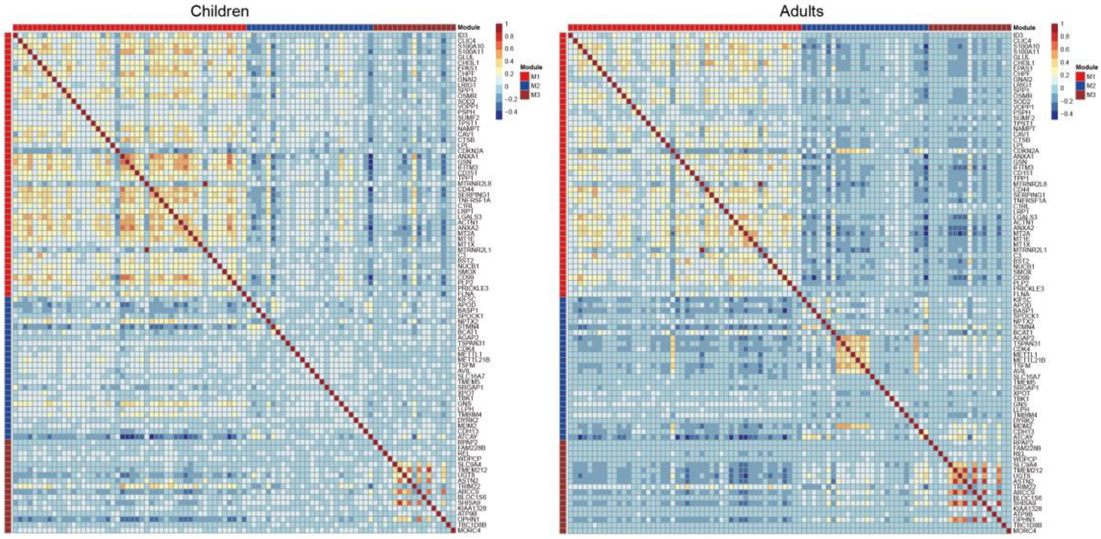

Supplementary Figure 5. The correlations of expression levels of any gene pairs in each module in data from children (left) and adults (right) with GBM.

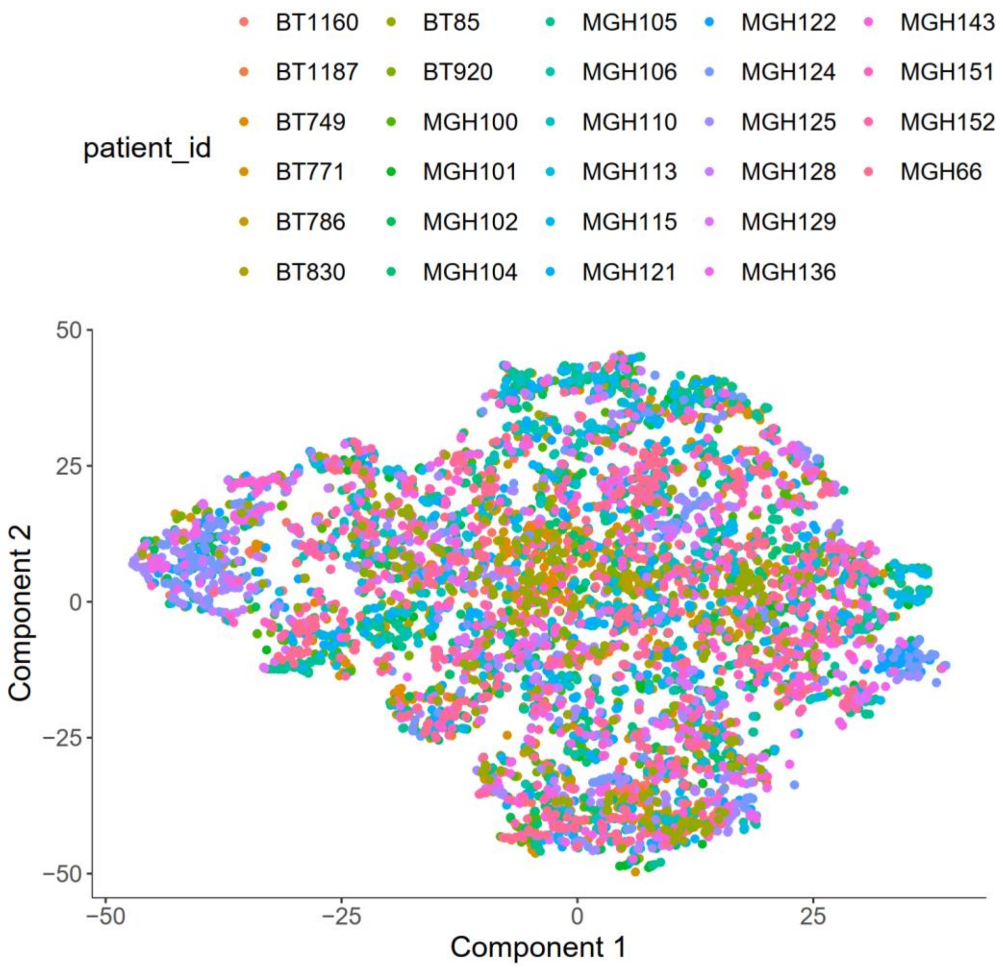

Supplementary Figure 6. The scores in each cell cluster. Boxplots showing the M2 (left) and M3 (right) scores of each cell cluster identified by our previous work using the same data.

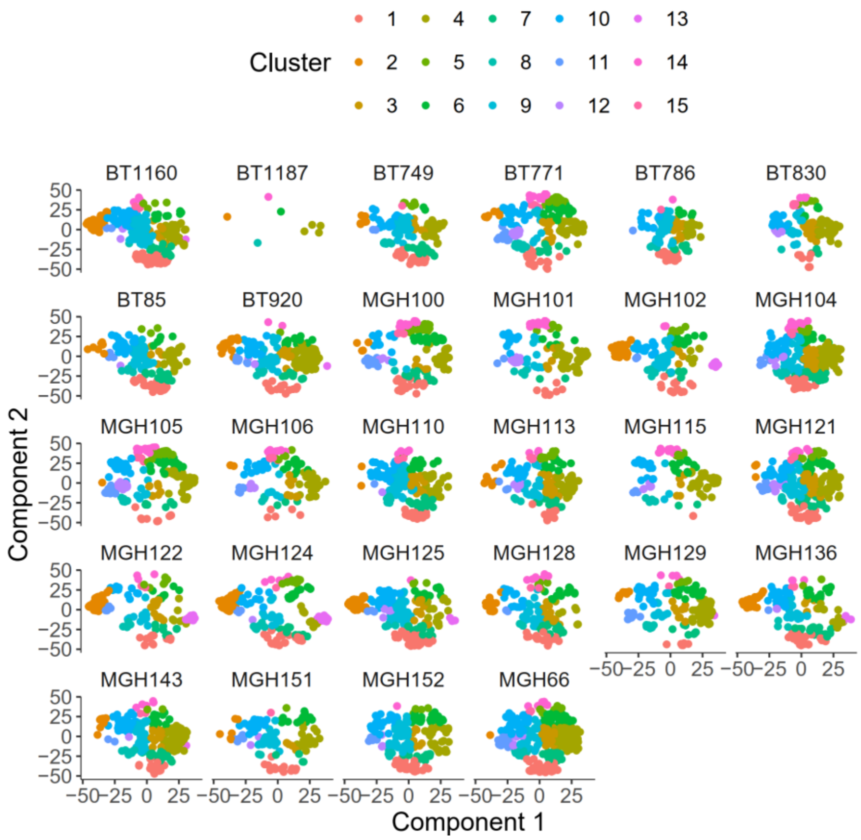

Supplementary Figure 7. The distributions of 15 cell clusters in each patients in data from Cyril et al. The tSNE plots were performed by Monocle. Each plot represents the cells from each patient, which were colored by clusters.

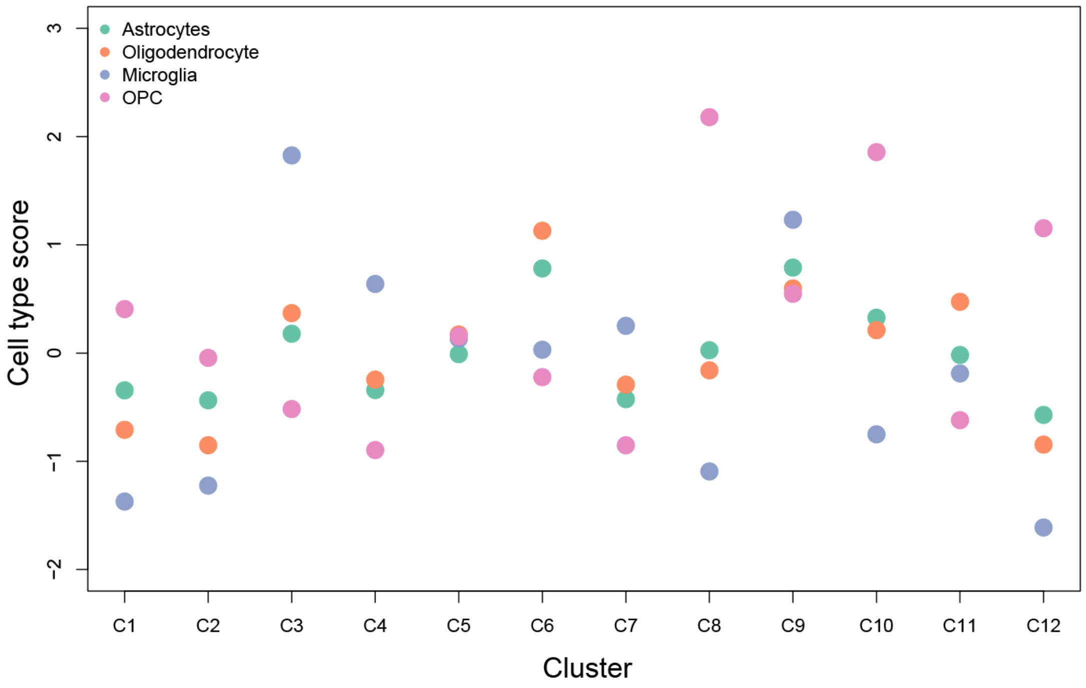

Supplementary Figure 8. The brain cell type scores of each cell cluster calculated using data from Darmanis et al.
